# Comparative Chemico-Physical Analyses of Strain-Free GaAs/Al_0.3_Ga_0.7_As Quantum Dots Grown by Droplet Epitaxy

**DOI:** 10.3390/nano10071301

**Published:** 2020-07-02

**Authors:** Inah Yeo, Doukyun Kim, Kyu-Tae Lee, Jong Su Kim, Jin Dong Song, Chul-Hong Park, Il Ki Han

**Affiliations:** 1 Dielectrics and Advanced Matter Physics Research Center, Pusan National University, Busan 46241, Korea; kim_do@naver.com (D.K.); cpark@pusan.ac.kr (C.-H.P.); 2 Nanophotonics Research Center, Korea Institute of Science and Technology, Seoul 02792, Korea; ktlee@gatech.edu; 3 Department of Physics, Yeungnam University, Gyeonsan 38541, Korea; jongsukim@ynu.ac.kr; 4 Post-Silicon Semiconductor Institute, Korea Institute of Science and Technology, Seoul 02792, Korea; jdsong@kist.re.kr

**Keywords:** droplet epitaxy, strain-free quantum dot, TEM-EDS

## Abstract

We investigate the quantum confinement effects on excitons in several types of strain-free GaAs/Al${}_{0.3}$Ga${}_{0.7}$As droplet epitaxy (DE) quantum dots (QDs). By performing comparative analyses of energy-dispersive X-ray spectroscopy with the aid of a three-dimensional (3D) envelope-function model, we elucidate the individual quantum confinement characteristics of the QD band structures with respect to their composition profiles and the asymmetries of their geometrical shapes. By precisely controlling the exciton oscillator strength in strain-free QDs, we envisage the possibility of tailoring light-matter interactions to implement fully integrated quantum photonics based on QD single-photon sources (SPSs).

## 1. Introduction

Photons appear to be ideal resources for implementing quantum bits (or qubits) in long-distance quantum communication and information processing [1]. Quantum photonic networks comprise interacting nodes that distribute entangled photons (qubits) through coherent optical links. On-demand entangled-photon-pair sources could realize scalable, all-optical quantum networks [1,2,3,4] and near-perfect bright, pure, and indistinguishable sources of QDs could realize highly scalable quantum networks [2,4,5,6]. However, precise QD engineering (e.g., 3D geometric and compositional profiles) is required to sustain such a high capability of quantum networking. Several investigations have reported the fabrication of strain-free QDs using droplet epitaxy (DE) [7,8,9,10,11,12,13,14] and by nanohole etching and infilling [15,16,17,18,19]. In the DE mode, QDs such as GaAs are crystallized by diffusing the initial liquid Ga droplets under an As flux, which avoids strain accumulation. Strain-free DE QDs can be implemented on an optical chip containing controlled shapes and sizes [4,10,12,14,20,21], thereby serving as high-fidelity sources of entangled photons [4,5,22,23]. However, low-level Al defects and Al intermixing have been probed to remain in strain-free GaAs DE QDs [11,13,24]. Atomic scale analyses have confirmed a low degree of Al defects in two-dimensional (2D) [11,25] and 3D composition profiles [13]. These Al remnants modify the electronic and phononic band structures of the GaAs DE QDs, degrading the fidelity of the entangled photons. As the entanglement fidelity relies on the minuscule energy splitting of fine-structure, corresponding quantum confinement corrections on QD band structures can substantially vitiate the quality of entanglement.

Herein, we have therefore investigated 3D quantum confinement characteristics of excitons in four series of strain-free GaAs/Al${}_{0.3}$Ga${}_{0.7}$As DE QDs grown by molecular beam epitaxy (MBE). By leveraging atomic-resolution energy-dispersive X-ray spectroscopy (EDS), we have systematically resolved 2D in-depth composition profiles of these individual samples. Using a 3D envelope-function model, we demonstrate precise control of the quantum confinement by capitalizing on the geometrical shape asymmetries in several experimental composition depth-profiles of DE QDs. We evaluate the individual quantum mechanical corrections to QD exciton states and their oscillator strengths using realistic potential profiles for the Al-interdiffused QDs. Additionally, we investigate the influence of anisotropic confining potentials on the quantum confinement properties of DE QDs to precisely control the exciton fine structures and the light-matter interactions precisely. Such precise engineering of strain-free QDs is critical for their implementation as scalable solid-state qubits on an optical chip.

## 2. Material and Methods

### 2.1. Growth of GaAs DE QDs

**Figure 1:**
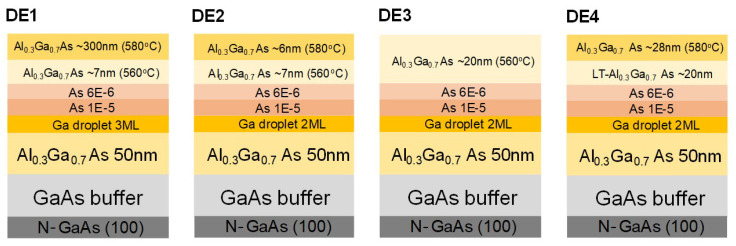
Schemat ics of layer structures and growth parameters in the four types of DE QDs.

Four species of GaAs QDs were grown by MBE using DE techniques [8,9,10]. The liquid Ga metallic droplets for the GaAs DE QD growth were formed initially in an ultra-high vacuum chamber (which maintains the partial pressure of As ($P_{\mathrm{As}}$) below ∼2 × 10${}^{-12}$ Torr prior to the Ga injection). We fabricated the liquid Ga droplets as Ga monolayers (MLs) deposited on an Al${}_{0.3}$Ga${}_{0.7}$As/GaAs substrate: two MLs for DE2, DE3 and DE4, and three MLs for DE1. The Ga droplets were crystallized into the GaAs QDs under a fixed As${}_{4}$ beam equivalent pressure (BEP) of 1.0 × 10${}^{-5}$ Torr. After a crystallization time of 60 s, we reduced the As${}_{4}$ BEP to 6.0 × 10${}^{-6}$ Torr and maintained the crystallization process for 720 s. The extended crystallization period enabled further structural shaping and improved the thermal stability. The GaAs DE QD islands were then protected by individual Al${}_{0.3}$Ga${}_{0.7}$As layers deposited at different temperatures under the same As${}_{4}$ BEP (6.0 × 10${}^{-6}$ Torr). The DE1 and DE4 GaAs nanostructures were covered by an 8-nm-thick and a 32-nm-thick Al${}_{0.3}$Ga${}_{0.7}$As layer, respectively, grown at substrate temperatures of ∼560 ${}^{\circ }C$ and ∼580 ${}^{\circ }C$. The DE2 and DE3 specimens were protected by a 20-nm-thick Al${}_{0.3}$Ga${}_{0.7}$As layer grown at near room temperature, followed by an 8-nm-thick and a 22-nm-thick layer of Al${}_{0.3}$Ga${}_{0.7}$As respectively, in the chamber while increasing the substrate temperature up to ∼560 ${}^{\circ }C$ (HT). While increasing the substrate temperatures up to ∼580 ${}^{\circ }C$, the DE1 and DE2 specimens were additionally protected by a 292-nm-thick and a 6-nm-thick Al${}_{0.3}$Ga${}_{0.7}$As capping layers, respectively, at a high substrate temperature (∼580 ${}^{\circ }C$). As evidenced by the capping thicknesses, capping time for DE1 was 50 times greater than that for DE2). The growth parameters of the four DE QD species are detailed in Figure 1. The specimens were subjected to a post-growth rapid thermal annealing (RTA) at ∼850 ${}^{\circ }C$ for 240 s in the standard RTA equipment to control the stoichiometry of the GaAs QD islands. Optimization of the ex situ thermally induced recrystallization process is described in the literature [10].

Notably, previous studies have fabricated ring-shaped nanostructures under similar crystallization conditions (i.e., 1.0 × 10${}^{-5}$ Torr; As supply to Ga droplets at ∼200 ${}^{\circ }C$) [26,27]. Slightly different parameters in a different MBE system yielded low-density DE QDs of 2–4/μm${}^{2}$. Previously, an identical fabrication of 2-ML Ga at ∼220 ${}^{\circ }C$ yielded high-density Ga droplets in previous studies [8,12,20,26]. In situ annealing at high temperatures (greater than 500 ${}^{\circ }C$) appears to considerably modify the structural morphology considerably and reduce the QD density [28,29].

### 2.2. TEM and EDS Analyses

Four individual cross-sectional TEM samples were prepared using the FIB technique. The corresponding four types of strain-free GaAs/AlGaAs DE QDs were resolved on the atomic level using a Titan 80-300${}^{\mathrm{TM}}$ TEM operating at 300 kV. Comparative chemico-physical characterizations of the QDs were performed using an analytical Talos F200X TEM microscope operated at 200 kV. We performed TEM-EDS elemental mapping and line-scanning on the FIB-prepared cross-sectional specimens. Owing to a compromise between a quantitative compositional precision and sample damage, we prepared sample specimens with thicknesses less than 50 nm. Note that too thin, crystalline specimens become sensitive to structural damage, charging, or the surface migration of contaminants. During the several minutes of data collection, the quantitative accuracy of a 2D elemental map declines due to inevitable spatial drift and damage to the thin samples. After 10 min of data acquisition, the Al compositional contrast across the QDs decreased by 50–75%. To improve the quantification accuracy, we selected a faster line-scanning mode and characterized the in-depth element profiles of the QDs across their central regions in the growth direction [001].

Thus, we achieved a spatial resolution of 1.6 Å in the scanning transmission electron microscopy mode. The data sets include the errors of averaging over the finite-width scanning strip (e.g., 20 nm). Compared to the theoretical composition of 50%, the average As composition was 52.3%, with a standard deviation of 1.8%. Our EDS analysis has an analytical accuracy of ∼2%.

## 3. Results and Discussion

**Figure 2:**
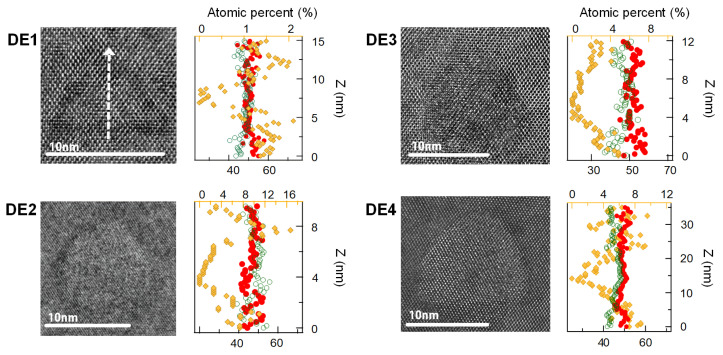
High-angle annular resolution dark-field-TEM images (gray) of the four GaAs DE QD species studied and the corresponding line concentration profiles of Ga (green open circles), As (red circles), and Al (yellow diamonds) obtained by EDS. The white arrow in the TEM image of DE1 represents the elemental line scan across the central region of each QD. The EDS data provide the relative amounts of atomic elements, and the experimental compositional errors for Ga and As were 1.4% and 2.3%, respectively, with corresponding standard deviations of 1.0% and 1.8%. The atomic percentage of Al is indicated along the top (yellow) axis and those of Ga and As are represented along the bottom axis.

In principle, DE QDs possess a deterministic and uniform composition profile with negligible intermixing. The layer structures of the four types of DE QD samples studied in the present work are shown schematically in Figure 1. Using transmission electron microscopy (TEM) EDS, we determined the corresponding chemical-composition profiles (in atomic percent) of cross-sectional specimens of embedded GaAs QD islands (cf. Figure 2). The in-depth profiles of the chemical constituents were resolved distinctly in the individual DE QD species (Figure 2). We characterized the chemical profiles $C_{\mathrm{Al}}$ of Al in the DE1, DE2, DE3, and DE4 specimens using their distinctive average slopes $|\frac{\partial C_{\mathrm{Al}}}{\partial z}{|}_{\Delta z<3\mathrm{nm}}$ of ∼0.6%/nm, ∼11.5%/nm, ∼2.3%/nm, and ∼1.8%/nm, respectively. Herein, we averaged the absolute first derivatives of second-degree polynomial fits over the upper and lower regions, $\Delta z=|z_{\mathrm{center}}-z|$ < 3 nm in the Al-composition profile of each line scan. The local Al concentration was particularly prominent in the top facet of QD DE2. We observed similar Al profiles in the EDS line scans of a few other QDs in the same specimen. Such crowns arise from the different diffusivities of the Ga and Al atoms on the side facets during capping [13,30]. Additionally, the Al enrichment beneath the QDs is attributable to the recrystallization of Al atoms dissolved in the liquid Ga droplet in the dots.

Bocquel et al. [13] retrieved the local 3D concentration profile of Al via atomic probe tomography (APT) on sharp-tipped samples embedding DE QDs (with a radius of a few tens of nm). In the present analysis, TEM EDS facilitated comparative chemical studies in different DE QD structures by enabling us to scan a broader cross-section of the sample (by approximately two orders of magnitude) than is possible using APT. Keizer et al. [30] analyzed the 2D inter-diffusion profile of Al in a cross-section of a DE QD specimen using scanning tunneling microscopy. Our line-scan analysis probed the in-depth elemental profiles of the DE QDs primarily by measuring the energies of their characteristic X-ray peaks. Limited by inevitable position drifts and damage to the thin samples, we found the average Al profiles from the EDS line scans to be less than 15 atomic percent at the QD boundaries. On a 20-nm-wide scanning strip of the Al${}_{0.3}$Ga${}_{0.7}$As host material, the average Al composition was ∼14.5% with a standard deviation of ∼2.0%.

The individual Al gradients result in different quantum confinement characteristics in the DE QD band structures. We calculated the quantum corrections to the QD exciton energies $E_{X}$ using a 3D envelope-function model in the k·p perturbation theory [31]. The QD energy of a single electron–hole pair is the sum of the GaAs bulk bandgap $E_{\mathrm{Gap}}$, the sub-band energies $E_{\mathrm{Sub}}$ of the carriers, and the direct Coulomb interaction energy *J*:

$$E_{X}=E_{\mathrm{Gap}}+E_{\mathrm{Sub}}-\left|J\right|.$$

Here, the latter two terms $E_{Q}=E_{\mathrm{Sub}}-\left|J\right|$ denote the quantum confinement characteristics of an electron–hole system in the QD. To calculate multi-particle energies, the exchange–correlation energy of the carriers also has to be taken into account [32,33]. In the excitonic complexes in large QDs, the interparticle correlations result in their nonseparable dynamics as well as an additional shift toward a higher binding energy [34]. We solved the following Schr$\ddot{o}$dinger equation numerically in 3D to evaluate the lens-shaped QD wavefunctions and energy eigenstates:

$$-\frac{\hbar^{2}}{2m\times (r)}\frac{\partial^{2}}{\partial^{2}r}\psi \left(r\right)+V\left(r\right)\psi \left(r\right)=E\psi \left(r\right).$$

In a finite-difference scheme, we evaluated the Coulomb energy *J* projected onto the exciton state $|\psi_{e}\psi_{h}\rangle$ using the integral [32] $J=-e^{2}\sum_{\sigma ,\sigma^{\prime }}\int \int d^{3}r^{\prime }d^{3}r\times \frac{\psi {*}_{e}\left(r,\sigma \right)\psi_{e}\left(r,\sigma \right)\psi {*}_{h}\left(r^{\prime },\sigma^{\prime }\right)\psi_{h}\left(r^{\prime },\sigma^{\prime }\right)}{\epsilon \left(r,r^{\prime }\right)\left|r-r^{\prime }\right|}$, for the dielectric screening $\epsilon (r,r^{\prime })$ at a given position r with spin σ. The material parameters used in this calculation can be found in the literatures [11,35].

**Figure 3:**
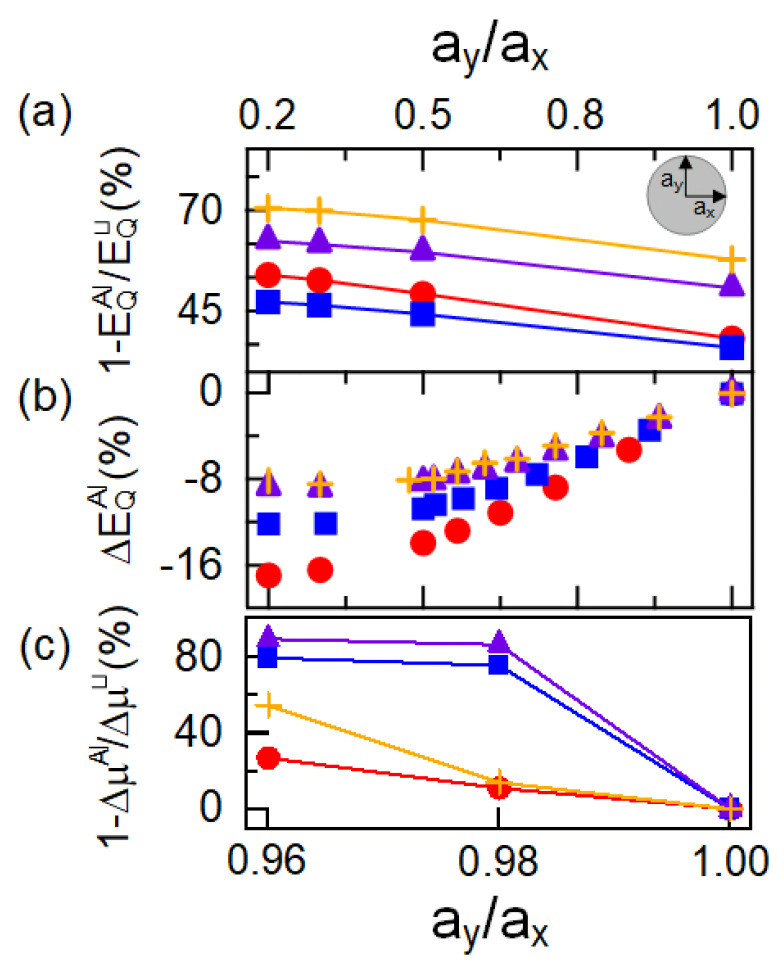
(**a**) Influence of Al interdiffusion $\Delta E_{Q}^{\mathrm{Al}}/E_{Q}^{⊔}$ and the quantum corrections in (**b**) the confinement energy $\Delta E_{\;Q}^{\mathrm{Al}}\left(=\Delta E_{Q}^{\mathrm{Al}}\left(a_{y}/a_{x}\right)/\Delta E_{Q}^{\mathrm{Al}}\left(1\right)-1\right)$ and (**c**) the fine-structure splitting $\Delta \mu^{\mathrm{Al}}/\Delta \mu^{⊔}$ in percent, on the asymmetries of the confining potentials of the four DE QD species (DE1, circles; DE2, rectangles; DE3, triangles; and DE4, pluses).

We modeled the confinement potentials in the DE QDs realistically by inserting the experimental mole fraction $x(z_{\mathrm{QD}})$ of Al into an empirical expression for the band gap [36] of $E_{{\mathrm{Al}}_{x}{\mathrm{Ga}}_{\left(1-x\right)}\mathrm{As}}\left(z_{\mathrm{QD}}\right)$ = 1.519 + 1.155*x* + 0.37$x^{2}$. We obtained the in-depth profile of $x(z_{\mathrm{QD}})$ from a least-squares polynomial fit of degree seven to the EDS Al composition data. Here, $z_{\mathrm{QD}}$ denotes the positions measured from the center of the QD along its growth axis. As the TEM-EDS allows us to analyze quasi-two-dimensional images and elemental maps of the QDs, we assume the lens-shaped QD radii $a_{x,y}$ and heights *h* to be (h,$a_{x,y}$) = (5, 15) nm, (6.5, 10) nm, (7.5, 13) nm, and (7.5, 17) nm, as extracted from the TEM images of QDs DE1-DE4, respectively. The randomness of the focused-ion-beam (FIB) sample preparation causes ambiguity in the cutting plane, which limits the positional precision of the geometric center of the QD. Table 1 summarizes the QD quantum confinement results, including the exciton oscillator strength f(= $\frac{E_{P}}{\hbar \omega_{0}}$$|\langle \psi_{h}|\psi_{e}\rangle {|}^{2})$ [37], for the four types of DE QDs. Compared to the energies $E_{\mathrm{Sub}}^{C/V}\left(⊔\right)$ and J(⊔) of pure GaAs QDs, the quantum mechanical corrections $E_{Q}$ range from 35% to 58% for the four individual species of Al-interdiffused QDs (cf. Figure 3a). When the anisotropy of a large QDs is precisely controlled to within ∼13% and ∼25% [21], the non-identical compositional profiles result in, respectively, 2.2–5.2% and 8.1–16.9% of the quantum confinement correction in $E_{Q}^{\mathrm{Al}}$. The individual Al composition profiles may lead to a FSS anomaly in the monotonically decreasing energy dependence [21]. The exciton oscillator strengths are tailored to *f*∼16, within a 4% average deviation, depending on the profiles of their realistic confining potentials.

The QD shape anisotropy $a_{y}/a_{x}$ can be controlled with careful growth conditions [10,21]. We also analyzed possible different shape anisotropies of the confinement potential relative to $a_{y}/a_{x}$. Increasing the in-plane asymmetry $a_{y}/a_{x}$ decreases the electron–hole sub-band confinement energy but increases the oscillator strength. Our 3D calculations demonstrate a noticeable difference in the asymmetry-dependence of $E_{Q}^{\mathrm{Al}}$ relative to that of pure QDs. The individual confining potentials of anisotropic QDs result in additional quantum corrections $E_{Q}^{\mathrm{Al}}/E_{Q}^{⊔}$ ranged from 11% to 16% at an in-plane asymmetry $a_{y}/a_{x}$ = 0.25 (cf. Figure 3a). The quantum correction $E_{Q}^{\mathrm{Al}}$ to the individual Al-interdiffused QDs has varied from 8% to 17% at the non-uniform quadratic confining potential profiles of $a_{y}/a_{x}$ = 0.25 (cf. Figure 3b). Compared to time-consuming, multimillion-atom simulations, the envelope-function model has been conventionally used to calculate the quantum-confinement energy in semiconductor QDs. Referring to the two-direction symmetries of zinc-blende materials, we note that the k·p calculations ignore the influence of the atomic symmetry [33]. However, the pseudopotential model characterizes both the geometric and atomic symmetry factors. We obtained increases in f up to approximately 7% by controlling the axial ratios precisely within the range of 0.25–1 for the four types of Al-interdiffused QDs. Such an engineered QD can enable accurate control of the light-matter couplings led by the marked quantum-confinement effects.

## 4. Conclusions

In this study, we have systematically analyzed four species of GaAs/Al${}_{0.3}$Ga${}_{0.7}$As DE QDs. We resolved the individual Al-composition gradients in the different strain-free QDs via atomic-resolution TEM EDS. Using a 3D envelope-function model for the DE QDs, we evaluated significant quantum mechanical corrections ranging from 20% to 45% due to the different band structures of the Al-interdiffused QDs. Controlling the shape anisotropy of the DE QDs increased the exciton oscillator strength up to approximately 7% for axial ratios in the range of 0.25–1. Such QD engineering is crucial for exploiting state-of-the-art qubit control based on DE QDs and their photonic networks on a chip.

## Figures and Tables

**Table 1:** Calculated quantum confinement characteristics of the four species of GaAs/Al${}_{0.3}$Ga${}_{0.7}$As DE QDs.

Type	DE1	DE2	DE3	DE4
$E_{\mathrm{Sub}}^{C}$[meV]	141.8	123.4	113.5	112.8
$E_{\mathrm{Sub}}^{V}$[meV]	41.5	32.5	33.6	33.4
|J|[meV]	36.8	32.9	29.8	29.6
*f*	15.4	16.0	16.4	16.4
$E_{\mathrm{Sub}}^{C}\left(⊔\right)$[meV]	100.1	92.3	70.5	61.9
$E_{\mathrm{sub}}^{V}\left(⊔\right)$[meV]	23.6	21.3	15.6	13.5
|J(⊔)| [meV]	33.0	32.1	28.1	26.1
f(⊔)	16.6	16.8	17.4	17.7

## Abbreviations

The following abbreviations are used in this manuscript:

DE	droplet epitaxy
QDs	quantum dots
SPS	single-photon sources
MBE	molecular beam epitaxy
EDS	energy-dispersive X-ray spectroscopy
TEM	transmission electron microscopy
APT	atomic probe tomography
FIB	focused-ion-beam
